# Reconstructing mental representations of block towers: A new bias for physical stability in visual working memory

**DOI:** 10.3758/s13423-026-03002-6

**Published:** 2026-09-29

**Authors:** Stefan Uddenberg, Jun Kwak, Brian J. Scholl

**Affiliations:** 1 Department of Psychology, University of Illinois Urbana-Champaign, Champaign, IL USA; 2 Department of Psychology, Yale University, New Haven, CT USA; 3 Wu-Tsai Institute, Yale University, New Haven, CT USA

**Keywords:** Intuitive physics, Visual working memory, Physical stability, Block towers

## Abstract

Recent studies have explored the perception of physical properties (such as mass and stability) in psychology, neuroscience, and artificial intelligence (AI), and perhaps the most popular stimulus from such studies is the *block tower –* since such displays (of stacked rectilinear objects) evoke immediate vivid impressions of physical (in)stability. Previous work has studied how people make higher-level decisions about such towers, often with explicit questions (“Which way will it fall?”), and limited to those tower properties that are systematically manipulated as explicit independent variables. Here, in contrast, we explored how such stimuli are spontaneously encoded into visual working memory in the first place. Using a novel drag-and-drop reconstruction task in a custom three-dimensional interface, observers briefly viewed randomly generated block towers, and then simply reproduced them from memory *–* without ever being asked about stability, per se. By simulating both the original and reconstructed towers in a physics engine, we discovered a novel bias: observers reliably remember towers as more stable than they originally were, even when the reconstructions are otherwise accurate. This reveals for the first time how visual memory is structured (and biased) not only by how scenes are segmented into objects, but also by how those objects are physically related to each other *–* all without ever asking anyone anything about physics.

**Supplementary Information:**

The online version contains supplementary material available at https://doi.org/10.3758/s13423-026-03002-6.

## Introduction

**Fig. 1:**
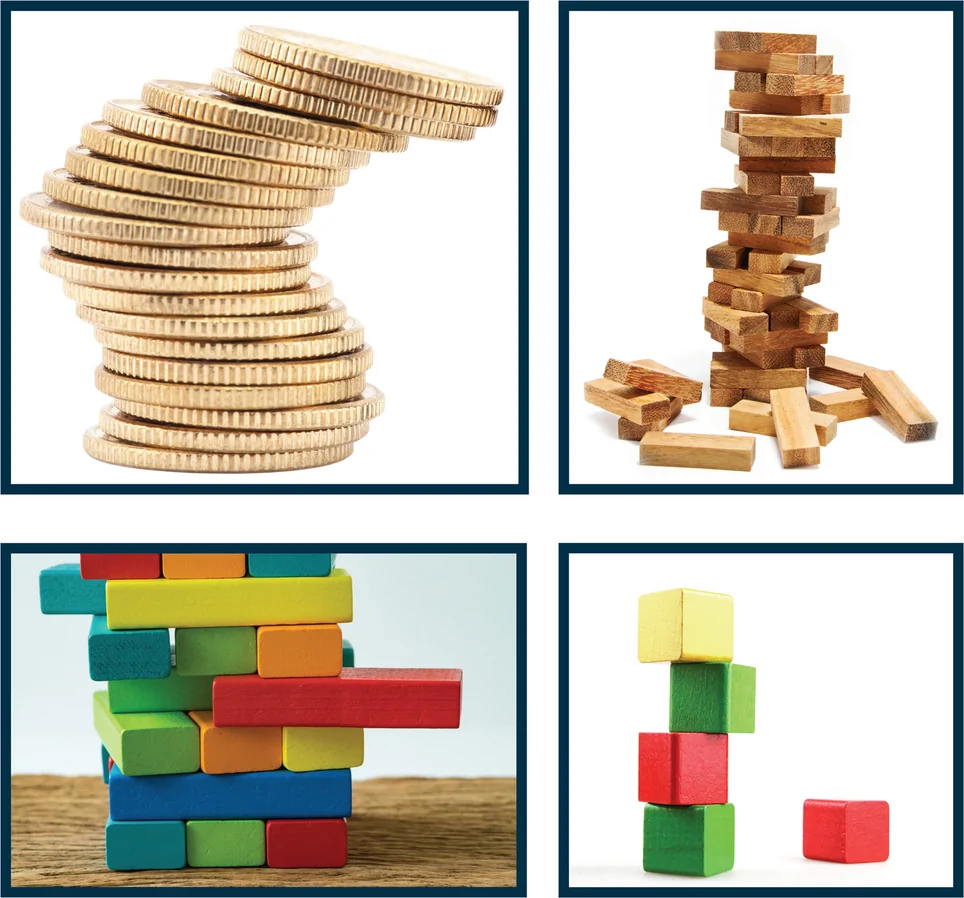
Familiar examples of physical (in)stability

Take a moment to look at the images in Fig. 1. What do you see? Of course you can appreciate the various low-level properties of those images, such as the colors and orientations of the various objects. But you may also readily perceive an even more obvious feature: each image depicts a stack of objects, evoking a vivid impression of physical (in)stability.

A vibrant and growing body of research in recent years has explored how we make judgments of physical stability when viewing such stimuli – including work in psychology (e.g., Battaglia et al., 2013; Hamrick et al., 2018; Mitko & Fischer, 2020), neuroscience (e.g., Fischer et al., 2016; Gallivan et al., 2014; Pramod et al., 2022; Schwettmann et al., 2019), and artificial intelligence (AI; e.g., Buschoff et al., 2025; Groth et al., 2018; Lerer et al., 2016; Sanchez-Gonzalez et al., 2018; Toussaint et al., 2018). And nearly all of this work explores human judgments of (in)stability in the most direct (and most explicit) way possible – by simply asking people overt questions about such displays, such as “Will this tower fall?”, “What direction will it fall in?”, or “How stable does this tower look, on a scale from 1 to 10?” (e.g., Battaglia et al., 2013).

### From judgment to memory

Existing work on intuitive physics has explored several possible underlying mechanisms for such stability judgments, such as the notion that we have a “mental game engine” that computes physical relationships to predict how dynamic scenes will unfold (Battaglia et al., 2013; Pramod et al., 2022; Ullman et al., 2017). But such proposals have mostly been agnostic or silent as to the roles of specific underlying cognitive processes in fueling such judgments, such as attention or spatial cognition (see also Firestone & Scholl, 2016; Mitko & Fischer, 2020).

Accordingly, the current work explores (for the first time, to our knowledge) how intuitive physics may structure – and bias – representations held in visual working memory (VWM). Of course, VWM does not operate over raw images, per se, but rather stores structured object-based representations. For example, it has been claimed that VWM capacity limits are sometimes determined not by the brute amount of to-be-remembered information, but by how that information is distributed among a number of discrete segmented objects (e.g., Adam et al., 2017; Luck & Vogel, 1997; Ngiam et al., 2023; Vogel et al., 2001; but see Oberauer & Eichenberger, 2013; for reviews, see Fukuda et al., 2010; Luck & Vogel, 2013; Mance & Vogel, 2013; Schurgin, 2018; Suchow et al., 2014; van den Berg et al., 2014). In this context, the present work seeks to push this sophistication even further: Might VWM be structured (and biased) not only by how scenes are segmented into discrete objects, but also by how those objects are physically related to each other?

### The current study: A novel reconstruction task

**Fig. 2:**
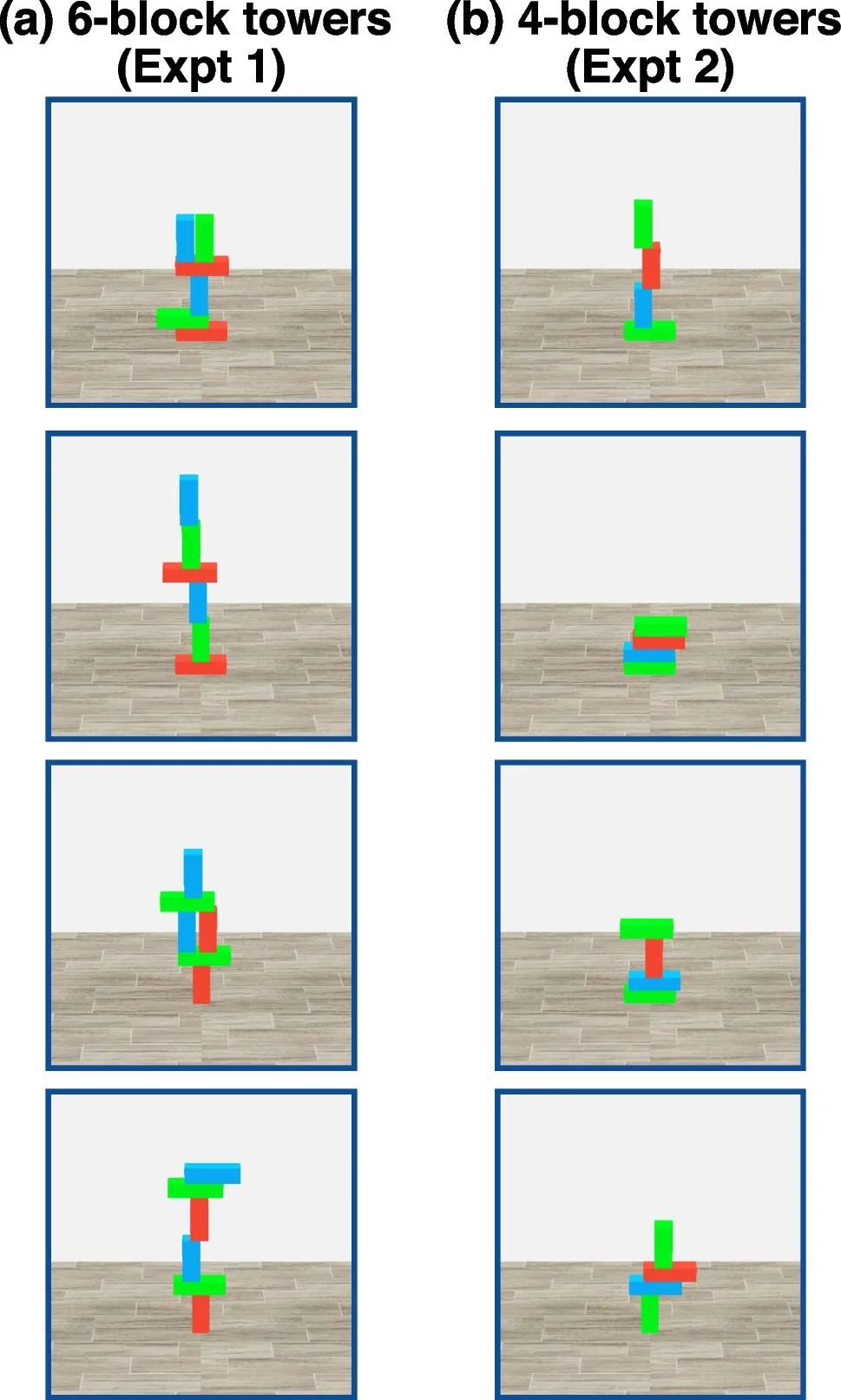
Examples of simulated block towers from our experiments: (**a**) six-block towers, from Experiment 1, (**b**) four-block towers, from Experiment 2

We explored interactions between physical stability and VWM using a novel reconstruction task. Observers first briefly viewed a randomly generated block tower (consisting of only a few colored blocks, as in the examples from Fig. 2).

**Fig. 3:**
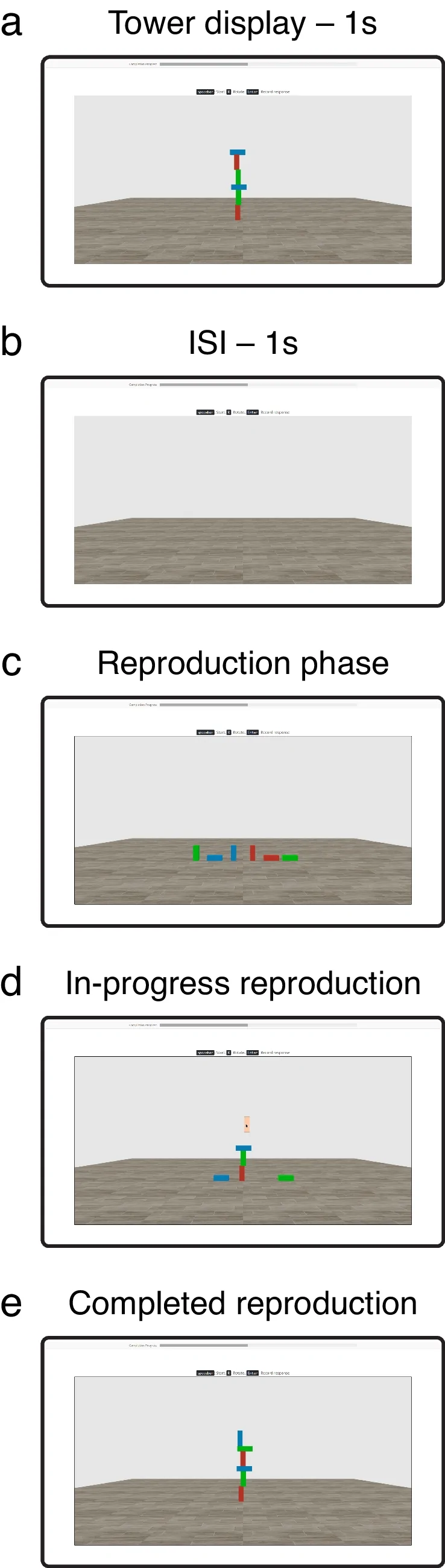
Depiction of the tower reconstruction task for a sample six-block tower. (**a**) The initial presentation. (**b**) A brief pause. (**c**) The reconstruction task begins with the component blocks all arrayed along the ground plane. (**d**) Here the subject is roughly halfway through reconstructing the tower, and is about to place the red block onto the blue block. (**e**) The subject’s final reconstruction

After that tower had disappeared, observers had to immediately reconstruct it from memory, using a novel virtual interface, with its constituent blocks initially spread out along the “floor” before them (as in Fig. 3).

This deceptively simple experimental method has two key advantages in the current context. First, whereas studies of explicit stability judgments must decide beforehand which relevant features to vary (e.g., the towers’ sizes, symmetries, shape envelopes, etc.), the current method has no such constraints: the towers are initially randomly generated, and in principle VWM for such stimuli could be biased in many different (multidimensional) ways. As such, simple reconstruction allows for data-driven insight, without needing to pre-specify the nature or extent of any memory biases. Second, this experimental paradigm can readily proceed without ever overtly mentioning physical stability – whereas such properties have been inextricably central to the explicit questions used in prior work. And as such, this task may have more generalizability, as it does not draw observers’ attention to features (e.g., stability) that they might not otherwise prioritize.

## Experiment 1: Visual working memory (VWM) for six-block towers

In this first test of VWM for physical stability, we employed the novel reconstruction task with images of towers randomly constructed from six blocks (as in Fig. 2a).

### Materials and methods

#### Participants

We recruited 100 US-based subjects (32 females; mean age = 30.27 years, *SD* = 10.37) using the Prolific Academic online labor market. (For a discussion of this pool’s nature and reliability, see Peer et al., 2017, 2021.) This sample size was preregistered, and was chosen arbitrarily to be in line with other recent studies of intuitive physics (e.g., Bi et al., 2025; Hamrick et al., 2018). Twelve additional subjects were excluded for one or more of the following preregistered reasons: reporting that they were inattentive during the experiment, as described in more detail below (three subjects); clicking away from the experiment window on one or more reconstruction trials (five subjects); stating that they had already participated in the study (three subjects); or having produced one or more towers that were less than four units tall (two subjects).

#### Apparatus

The experiment was conducted using custom software written using a combination of Python, Javascript, CSS, and HTML. Key libraries used included jsPsych (de Leeuw, 2015) for experiment presentation, and babylon.js (https://www.babylonjs.com) for three-dimensional (3D) scene creation. Subjects completed the experiment via a custom web page which could be loaded in any modern web browser on their own laptop or desktop computers. (Because our experiment required a relatively large display and the use of a computer mouse to make responses, mobile devices such as phones and tablet computers were explicitly disallowed, and attempts to access the experiment from such a device led to its immediate termination along with an error message.) The experiment window expanded to take up the full extent of the subject’s screen after they consented to participate. We also included a link to a simple custom browser compatibility check, allowing each subject the opportunity to ensure that their system was able to process the sorts of 3D scenes that would be shown.

#### Stimuli

The kinds of stimuli subjects saw and reproduced are depicted in Fig. 2a. Each randomly generated tower shown to subjects consisted of six colored blocks, with each block measuring 1 × 1 × 3 units in size. (Because subjects completed the experiment on their own browsers and displays, we report all stimulus dimensions in arbitrary units. On a typical 1,600 px × 900 px 15.6-in. laptop display viewed from 57 cm, each block subtended approximately 1.53° × 0.56° when horizontal.) The towers were constructed with blocks that could either be oriented horizontally or vertically, and were built up such that they were “locally stable” (so that whenever a new block was added to the tower, it would not fall off of its immediate support in isolation, based on the position of its center of gravity). Each of the 3D matte blocks were colored red ([RGB 0, 74, 54]), green ([0, 255, 23]), or blue ([10, 176, 255]), and cycled through those colors in that order as the tower was built up from the ground. The towers were shown in a simple virtual environment with a wooden textured floor and white walls (as in Fig. 3). The scene was lit with an ambient white light source aiming up and to the left with an intensity of 0.25, and two white point light sources placed around the space (XYZ coordinates: [−20, 20, −20] and [20, −20, 20], respectively). The camera was positioned approximately 13.6 lengthwise-blocks away from and above the block tower scene (XYZ coordinates: [0, 8, −40]), and was angled to face slightly above the scene’s origin (focused on the XYZ coordinate [0, 8, 0]).

#### Procedure

Subjects first gave their informed consent and agreed to answer open-ended questions, using a procedure designed to reduce subject attrition (Zhou & Fishbach, 2016). The experiment window then expanded to take up the full extent of the screen, and subjects read detailed written instructions for the task, as described below. On each of two trials, subjects were shown the empty virtual environment. After a keypress, a randomly generated block tower was displayed for 1 s (Fig. 3a). After another 1 s of again viewing the empty virtual environment (Fig. 3b), the tower’s constituent blocks reappeared on the virtual floor, equally spaced apart in a random order and in random orientations (horizontal or vertical; Fig. 3c). Subjects were instructed beforehand to reconstruct the tower that they just saw by clicking and dragging the blocks into position. They were able to change a block’s orientation between horizontal and vertical by pressing the “R” key on the keyboard while dragging it. When the subject released a block, it instantly dropped straight down until it collided with either the ground or with another block, at which point it stopped, remaining fixed in place regardless of its stability. Subjects adjusted the blocks as often (and for as long) as they wished, after which they pressed the spacebar to submit their reconstruction (which they were only allowed to do after moving at least five of the blocks from their initial starting positions). A sample in-progress reconstruction is depicted in Fig. 3d, and a sample final reconstruction is depicted in Fig. 3e.

Subjects were then asked a series of debriefing questions, including: (a) what they thought we were testing; (b) how well they thought they did in the reconstruction task, on a scale of 1 (terrible) to 100 (perfect); (c) whether they used any particular strategies in the task; (d) whether they interrupted their completion of the survey in any way (e.g., by refreshing the task page or switching tabs/windows); (e) whether any part of the procedure was unclear or if they encountered any problems during the experiment; (f) whether they had participated in the task before; and (g) how well they were able to pay attention over the course of the experiment, on a scale of 1 (very distracted) to 100 (very focused). After submitting their responses, they were redirected to Prolific for compensation, and the experiment ended.

To confirm that subjects reproduced the towers with sufficient fidelity, we performed a supplemental memory analysis comparing silhouette overlap between the initially presented and reproduced towers, compared to randomly permuted pairs of towers (included in tabs 14 and 15 of the Supplementary Data File, with details for both experiments).

### Results

We measured tower stability offline by simulating the influence of gravity for 10 s (600 frames) on both the towers initially shown to subjects (initial towers), and the towers that they reconstructed (reconstructed towers). Simulations were generated using the Panda3D game engine (https://www.panda3d.org/) in concert with the Bullet physics engine (https://pybullet.org/wordpress/), with each block assigned a mass of 1 unit and with gravity set to –9.81.

**Fig. 4:**
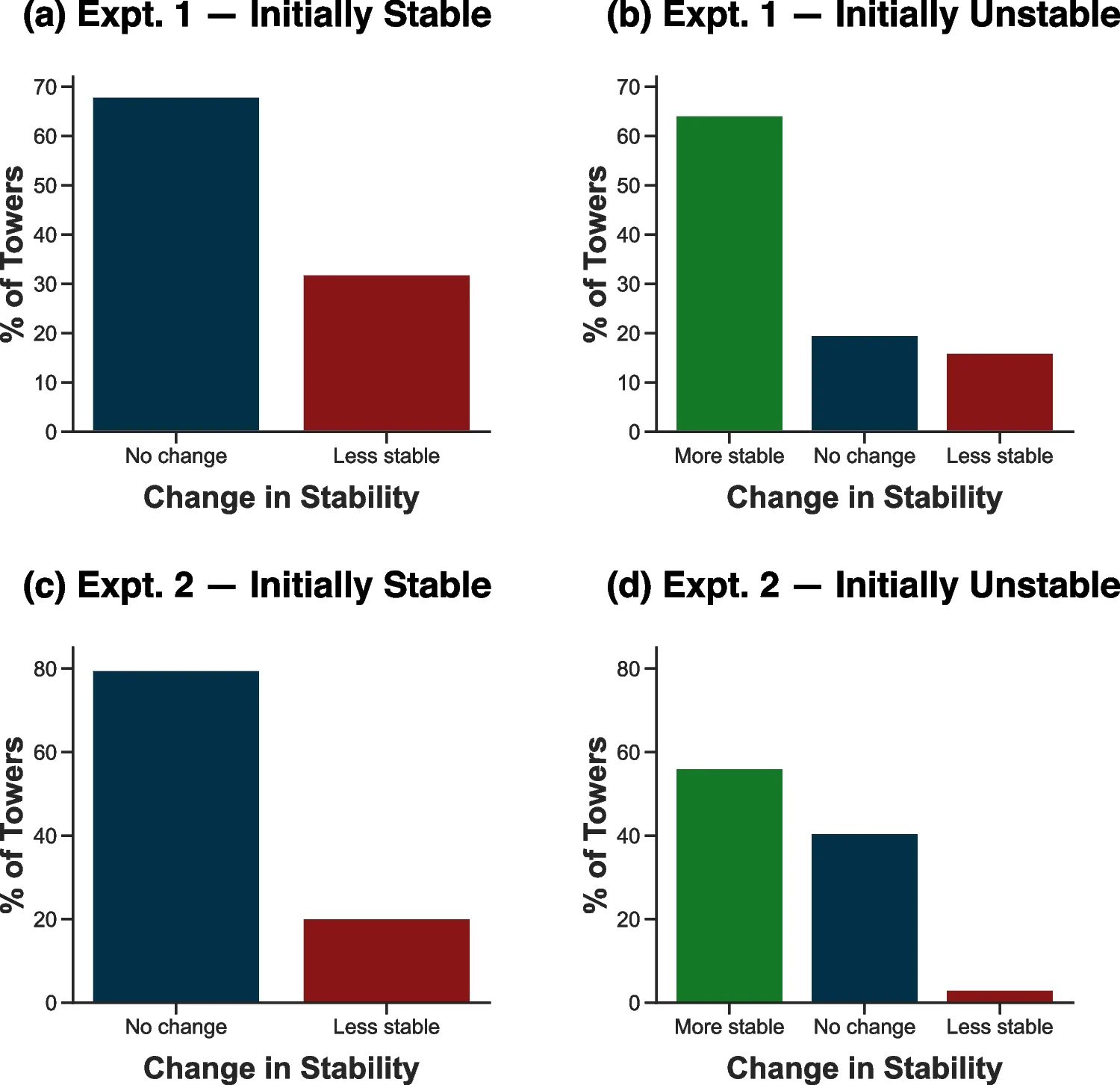
Categorical changes in stability in reconstructed towers (compared to initial towers) in Experiment 1 (**a** and **b**) and Experiment 2 (**c** and **d**)

Per our preregistered analysis plan, we first evaluated the stability of the initial towers and reconstructed towers categorically. For this purpose, we simply categorized a tower as stable if (during its subsequent simulation) no block moved more than 0.5 units of distance from its original starting location, or as unstable if at least one block moved more than this distance. And we further quantified instability in terms of the brute number of blocks that moved more than this distance (from 1 to 6). For stable towers (*N* = 144), we were thus able to measure how many reconstructions became less stable, and how many stayed stable. (Note that an initially stable tower can never become more stable via this initial categorical metric.) And for unstable towers (*N* = 56), we were able to measure how many reconstructions became less stable, how many became more stable, and how many remained at the same level of instability. The resulting percentages are depicted for initially stable towers in Fig. 4a, which reveals that most stable towers were reproduced as stable (by approximately a 2 to 1 ratio – 98 stable vs. 46 less stable – with this difference being highly reliable by a binomial test, *p* <.001). More theoretically consequential were the resulting percentages for the initially unstable towers, as depicted in Fig. 4b. Here we observed a striking and unexpected pattern, with reconstructed towers being reliably more stable than were the initial towers (as confirmed by a difference of observed proportions, as measured in terms of the raw counts – 36 more stable vs. 11 unchanged vs. 9 less stable – *Χ*^2^[2, *N* = 56] = 24.25, *p* <.001, ϕ = 0.66). This effect was also dramatic in its magnitude, with reconstructions becoming more stable four times as often as they became less stable.

**Fig. 5:**
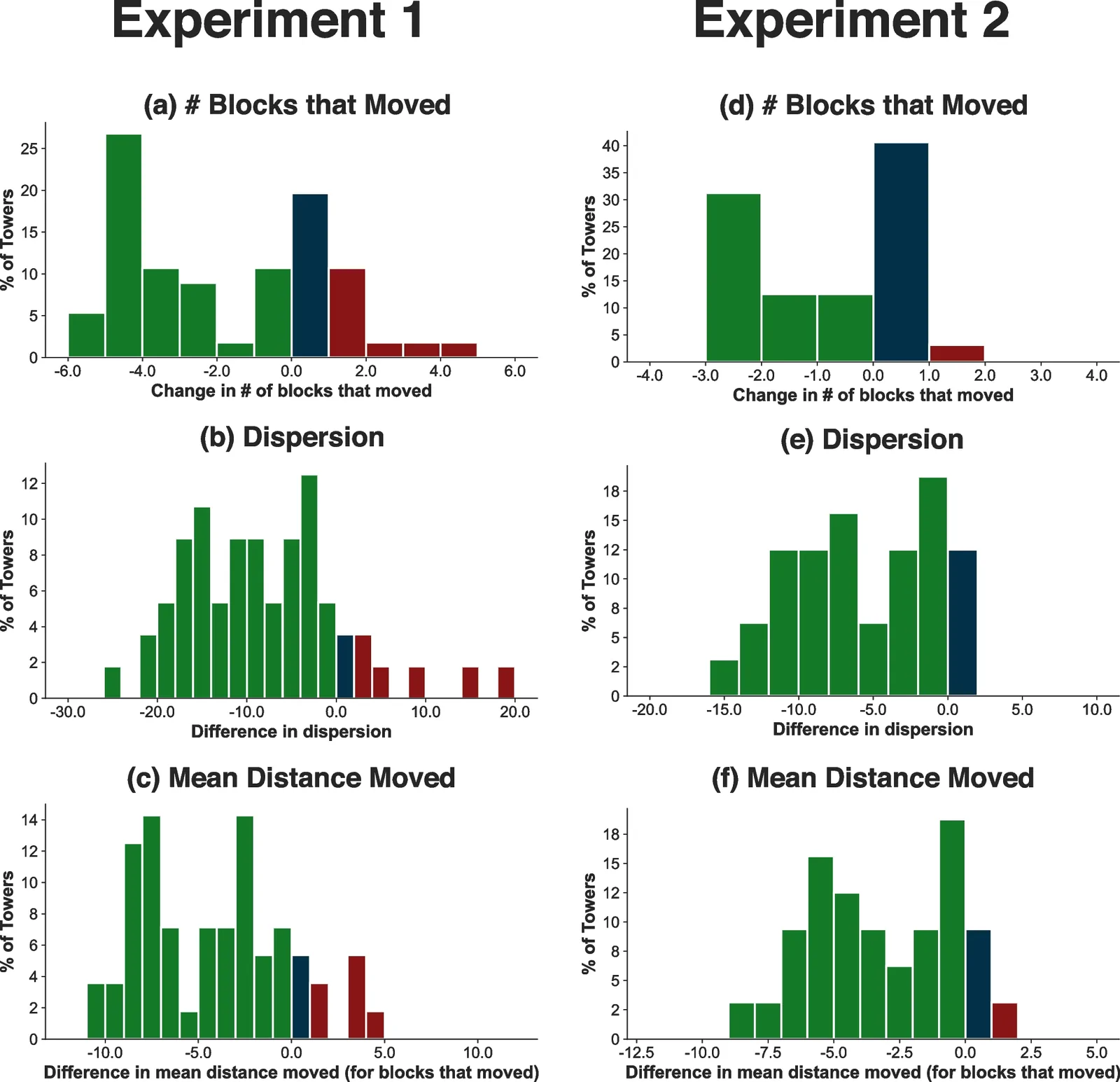
Histograms for changes between initial unstable towers and their reconstructions, for both Experiment 1 and Experiment 2, in terms of (**a** and **d**) number of blocks that moved, (**b** and **e**) the resulting dispersion, and (**c** and **f**) the average distance moved (for blocks that did move). Values that indicated greater stability in the reconstructed towers are colored green, values that indicated less stability are colored red, and a single bar closest to the same level of stability is colored blue

Beyond these categorical measures, we also analyzed the reconstructed towers in terms of three continuous measures of stability, each of which compared the initial scenes to the final post-simulation tableau. The first of these was the same metric used in the initial categorical analyses: the number of blocks that moved more than 0.5 units of distance from their original starting locations. In addition, we also measured both the dispersion of the blocks (calculated as the difference between the x-coordinates of the blocks that were furthest from one another in the final tableau) and the average distance that blocks moved (from the initial scene to the final tableau), when they moved at all (defined as moving more than 0.001 units of distance from their starting location). These three continuous metrics of (in)stability are depicted in Fig. 5 in terms of histograms for the changes between initial unstable towers and their reconstructions. In each case, values that indicated greater stability in the reconstructed towers are colored green, values that indicated less stability are colored red, and a single bar closest to the same level of stability is colored blue. Inspection of this graph reveals a clear and consistent pattern: for all three metrics, subjects reliably reconstructed towers as more stable than they initially were. This impression was confirmed with a series of *t*-tests: relative to the initial towers, reconstructions had fewer blocks move (Fig. 5a; *M* = –2.23, *SD* = 2.66; *t*(55) = 6.27, *p* <.001, *d* = 0.84), had less dispersion (Fig. 5b; *M* = –8.07, *SD* = 8.97; *t*(55) = 6.74, *p* <.001, *d* = 0.90), and had less average movement among those blocks that did move (Fig. 5c; *M* = –4.07, *SD* = 3.96; *t*(55) = 7.70, *p* <.001, *d* = 1.03).

## Experiment 2: VWM for four-block towers

Although each trial in Experiment 1 involved only a single tower, that tower was always composed of six different blocks, with this value being above most contemporary estimates on the limits of short-term memory (as in the “magic number 4”; e.g., Cowan, 2001). As such, the bias to reproduce towers as more stable than they originally were might have stemmed not from a memory bias per se, but rather from an inference about likely degrees of stability in the absence of a robust memory. To test this possibility, we replicated Experiment 1 but now using towers composed of only four blocks each – where this less demanding load was more comfortably within the range of typical memory capacity estimates.

### Materials and methods

This experiment was identical to Experiment 1, except as noted. We again recruited 100 US-based subjects (48 females; mean age = 32.06 years, *SD* = 11.48) using Prolific, with this preregistered sample size chosen to match that of Experiment 1. Ten additional subjects were excluded for one or more of the following reasons: reporting that they were inattentive during the experiment (five subjects); clicking away from the experiment window on one or more reconstruction trials (four subjects); or having produced one or more towers that were less than 3 units tall (two subjects). Each of the blocks now cycled through the colors green, blue, and red (in that order) as the tower was built up from the ground.

### Results

The percentages of towers that changed their categorical levels of stability are shown in Fig. 4, broken down by towers that were initially stable after simulation (with no blocks that moved; Fig. 4c) versus those that were initially unstable (with one or more blocks that moved; Fig. 4d). Inspection of Fig. 4c again shows that most stable towers (*N* = 168) were reproduced as stable (by approximately a 4 to 1 ratio – 134 stable vs. 34 less stable – with this difference being highly reliable by a binomial test, *p* <.001). Critically, we also replicated our original striking pattern for initially unstable towers (*N* = 32), as depicted in Fig. 4d: reconstructed towers were reliably more stable than were the initial towers (as confirmed by a difference of observed proportions, as measured in terms of the raw counts – 18 more stable vs. 13 unchanged vs. 1 less stable – *Χ*^2^(2, *N* = 32) = 14.31, *p* <.001, ϕ = 0.67). Now this effect was even more dramatic in its magnitude, with reconstructions becoming more stable 18 times as often as they became less stable.

Our three continuous metrics of (in)stability are depicted in Fig. 5, again in terms of histograms for the changes between initial unstable towers and their reconstructions. Inspection of this graph reveals the same clear and consistent pattern observed in Experiment 1: for all three metrics, subjects reliably reconstructed towers as more stable than they initially were. This impression was again confirmed with a series of *t*-tests: relative to the initial towers, reconstructions had fewer blocks move (Fig. 5d; *M* = –1.28, *SD* = 1.37; *t*(31) = 5.28, *p* <.001, *d* = 0.93), had less dispersion (Fig. 5e; *M* = –5.62, *SD* = 4.62; *t*(31) = 6.88, *p* <.001, *d* = 1.22), and had less average movement among those blocks that did move (Fig. 5f; *M* = –3.17, *SD* = 2.64; *t*(31) = 6.80, *p* <.001, *d* = 1.20).

## Discussion

**Fig. 6:**
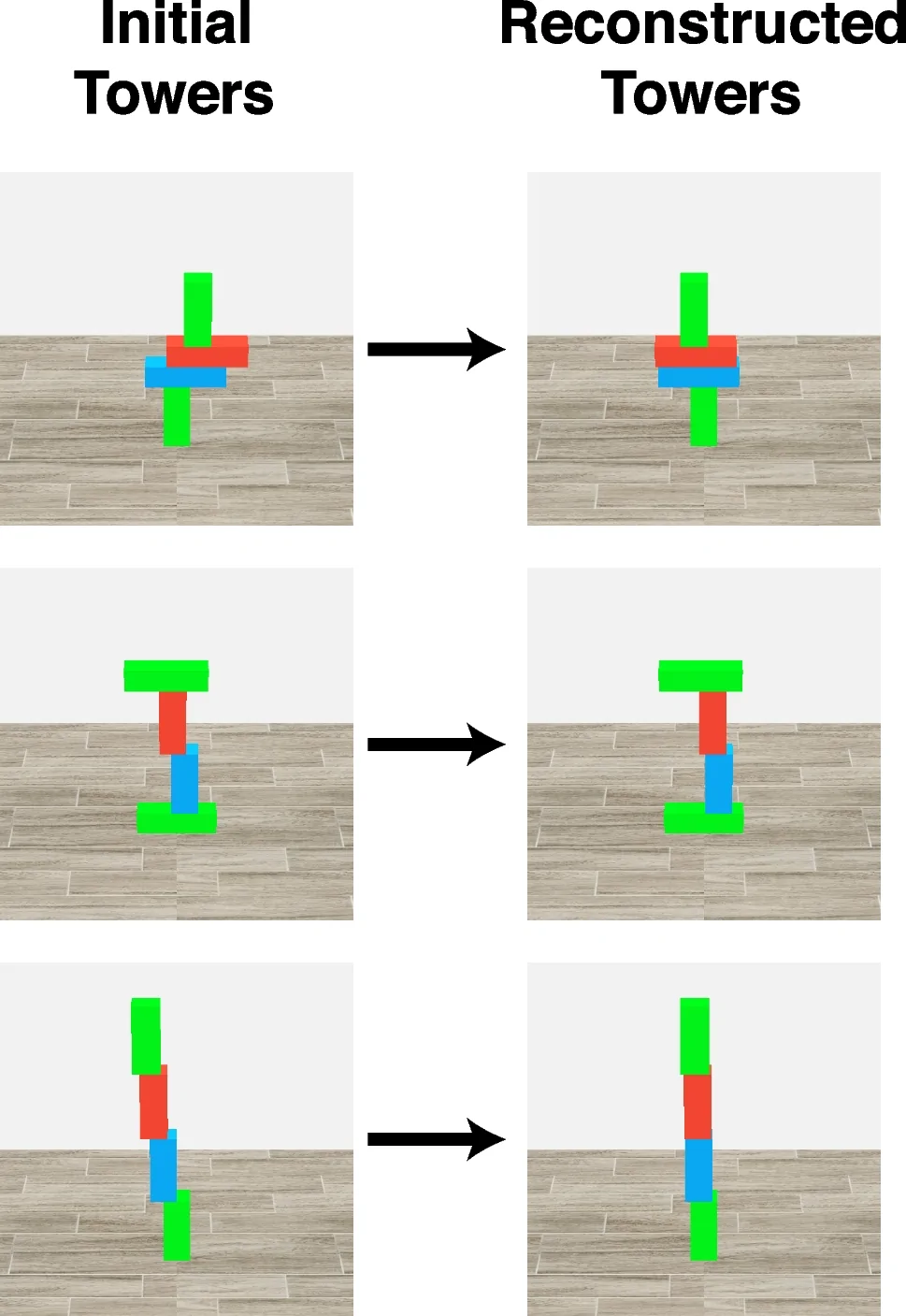
Examples of towers initially shown to subjects (left column) and subsequently reproduced as more stable (right column)

The central result of this study was a novel bias in VWM related to intuitive physics: observers reliably remembered block towers as more stable than they originally were, as measured in a reconstruction task. Beyond the statistical support for this conclusion as summarized in Figs. 4 and 5, we can also see several representative examples of this in practice in Fig. 6: in each case, the left column depicts an initial random tower shown to a subject, and the right column depicts their (more stable) reconstruction. This effect seemed especially robust in at least six related ways. First, the effect was highly statistically reliable, to a degree far below conventional significance thresholds – with the average *p*-value of the primary comparison across the experiments being *p* =.00039. Second, the magnitude of the primary comparison was also large, with observations in one of the experiments exhibiting this pattern by an 18:1 ratio. Third, we observed this same effect across many different measures of stability, including quantitative measures (such as the ultimate dispersion of the blocks after simulation). Fourth, this effect generalized to towers that both did and did not transcend typical estimates of VWM capacity. Fifth, this bias occurred despite subjects’ reproductions being overall highly accurate (as detailed in Tabs 14 and 15 of the Supplementary Data File for both experiments). Finally, this effect occurred despite subjects never being asked, told, or shown anything at all about the physical properties of the towers (in contrast to existing work in the published behavioral literature).

### A surprising outcome?

We were surprised by this memory bias. In fact, we initially predicted the opposite outcome – that unstable towers would be misremembered as *more* unstable, exacerbating a key salient physical property of the towers – for at least three reasons:

First, past work suggests that instability may be especially attention-grabbing: it can not only guide selective attention (Firestone & Scholl, 2016) and lead to higher levels of perceived “interestingness” of towers (Holdaway et al., 2021), but can even fuel a powerful visual search asymmetry: people are quicker to find less stable images among stable distractors (e.g., a tilted vase among upright vases) than stable images among less stable distractors (e.g., an upright vase among tilted vases; Yang & Wolfe, 2020).

Second, note that the memory bias we actually observed leads to the mitigation (or removal) of a distinctive visual feature (viz. instability), whereas past work has more often observed augmentation (or addition) of salient features. Perhaps the most famous and emblematic examples of this are caricature effects for faces, wherein exaggerated representations of a known person are more easily and quickly recognized than are veridical depictions (e.g., Lee et al., 2000; Rhodes, 1997). Such caricature effects may be a domain-general feature of perception (Sun et al., 2024), as they are found in many contexts including in the auditory domain (Whiting et al., 2020).

Third, salient features are also over-weighted in many other contexts, including the extraction of temporal summary statistics – as when looming circles bias estimates of mean size (Albrecht & Scholl, 2010), or more intense emotional expressions bias estimates of mean emotion (Goldenberg et al., 2022). In the face of such evidence, the robust demonstration of a bias for *greater* physical stability in memory seems especially striking.

### Advantages of reconstruction

This work also demonstrates the utility of our (deceptively simple) reconstruction task: by having observers simply view and reconstruct towers from memory, we were able to uncover a novel memory bias that had not been observed in previous work. This approach allowed us to avoid two major downsides of most prior studies of intuitive physics.

First, whereas past work most often asked subjects directly about physical properties (“Will it fall?”), this reconstruction task allowed us to (implicitly) ask about mental representations of physical stability, without ever (explicitly) asking about physical stability. Second, by having subjects freely reconstruct the towers, there was no need for us as experimenters to choose which features of the towers we would manipulate or measure in advance: both the initially presented and reconstructed towers could vary with relatively few constraints on how they were built.

Future work could use this same approach to explore the underlying perceptual roots of various other intriguing phenomena, such as the perception of relationships between objects (e.g., containment or attachment; Hafri & Firestone, 2021; Strickland & Scholl, 2015) or causal history (Chen & Scholl, 2016; Schmidt et al., 2016).

### Why?

Where does this memory bias come from? One deflationary possibility that our existing data can refute is simple familiarity. In principle, our findings could reflect a bias *for* stability rather than *towards* stability. That is, subjects may be predisposed to produce towers that are entirely, categorically stable – perhaps due to the ubiquity of such arrangements in everyday life, wherein unstable arrangements do not tend to stick around for long. (If you see a group of objects such as that in the upper left quadrant of Fig. 1, it will probably look different the next time you see it.) This account, however, predicts that initially unstable towers should typically be reconstructed as entirely stable. But this was not the case: subjects tended to reconstruct unstable towers as *more stable*, even when the result was still categorically *unstable*. This can be appreciated both from the roughly continuous distributions seen in the histograms of Fig. 5, and in Figs. S1 (depicting the changes in stability for each of the towers, across our continuous metrics of stability, for both experiments) and S2 (depicting the tremendous overall reproduction accuracy across both experiments) in Tabs 13 and 16 of the Supplementary Data File. Our results thus reflect a bias towards (but not categorically for) stability. Indeed, even if we limit our analyses solely to the most unstable towers, we can see that participants were quite willing to reproduce even the most initially unstable towers as very unstable – just a bit less so than in the original displays – thus ruling out the possibility that they were merely hesitant to produce unstable configurations (see Figs. S3 and S4 in Tabs 17 and 18 of the Supplementary Data File; these are analogous to Figs. 4 and 5 shown earlier in the paper, but focused only on the most initially unstable towers).

Another deflationary possibility is that participants were not “reconstructing” the towers, but merely “constructing” them anew – creating brand new towers cut from whole cloth, as if they had closed their eyes during the main task. However, our key analyses all hold up even when constrained to just those towers that were reproduced well, as operationalized by good silhouette overlap (from a side-on view) between the initially presented and reconstructed towers (see Tab #19 of the Supplementary Data File for full details of these analyses for both experiments).

A final alternative account might appeal to the possibility that participants reduced the complexity and details of the towers by aligning the blocks more, which would result in greater stability only as a “side effect” of a more general response tendency to produce simpler towers. However, our results hold even when we limited our analyses to those towers that showed no more alignment of the blocks in the reproductions (see Supplementary Data File Tab 20).

Having dispatched these deflationary possibilities, we can also identify at least three theoretically interesting reasons for the bias towards physical stability in visual working memory. One possibility is that it stems from an urge to *improve* or shore up the towers. If you saw the arrangement from the upper left quadrant of Fig. 1 on your desk, for example, your next move might be to manually align the coins by hand. That such an effect would emerge even when unnecessary (and even with virtual objects on a computer screen!) may speak to how ingrained such impulses can be. Another possibility is that the effect derives from memory biases of the local stability of each block, as in past findings of hierarchical encoding of visual ensembles (Brady & Alvarez, 2011). In effect, the global tendency towards more stability may be driven by ensemble coding of local stability – since the initial towers all had entirely locally stable configurations. Finally, it is intriguing to consider the possibility that people effectively have a prior expectation for stability – not in the form of a conscious belief, but rather as a type of spontaneous Bayesian prior in perception and memory. We know from past work, for example, that such priors can readily bias downstream reproductions in other contexts (Langlois et al., 2021; Uddenberg & Scholl, 2018). And these priors may have deep connections to the classic Gestalt notion of “Prägnanz” (Van Geert & Wagemans, 2023, 2024). Whichever of these (non-mutually exclusive) possibilities may lie behind the current results, this project demonstrates a new way in which two of the most active research areas in our field – visual working memory, and intuitive physics – may have unexpectedly rich connections.

## Supplementary Information

Below is the link to the electronic supplementary material.

Supplementary file1 (XLSX 2515 kb)

## Data Availability

The raw data for each experiment can be viewed in the Online Supplementary Data File for this study. Preregistrations for each experiment can be viewed at: https://aspredicted.org/FKX_7J7 (Experiment 1), and https://aspredicted.org/X7V_HJL (Experiment 2).

## Code availability

Analysis scripts can be viewed in the Online Supplementary Data File for this study.

## Declarations

### Open practices statement

The hypotheses, methods, and analysis plan were preregistered prior to data collection. Preregistration for Experiment 1 can be found at https://aspredicted.org/FKX_7J7. Preregistration for Experiment 2 can be found at https://aspredicted.org/X7V_HJL. There were no deviations from the preregistration. All study materials, primary data, and analysis scripts are available in the Supplementary Data File.
